# 

**Published:** 1878-11

**Authors:** 


Cyclopedia of the Practice of Medicine, edited by Dr. H. Von Ziemssen, Professor of Clinical Medicine in Munich, Bavaria. Vol. XVII.

General Anomalies of Nutrition and Poisons. Vol. XIII.

Diseases of the Spinal Cord and Medulla Oblongata. New York: Wm. Wood & Co.

A Treatise on the Science and Practice of Midwifery. By W. S. Playfair, M. D., F. R. C. P., with notes and additions, Robert P. Harris, M. D. H. Lea: Philadelphia. Buffalo: T. H. Butler.

A Practical Treatise on the Medical and Surgical uses of Electricity. By George M. Beard, A. M., M. D., and A. D. Rockwell, A. M., M. D. New York: Wm. Wood & Co.

The Cell Doctrine; its history and present state. By James Tyson, M. D. Philadelphia: Lindsay & Blakiston, 1878. Buffalo: T. H. Butler.

A Guide to the Practical Examination of Urine. By James Tyson, M. D. Philadelphia: Lindsay & Blakiston. Buffalo: T. H. Butler.

A Monograph on the Treatment of Diphtheria, a new Etiology and Pathology. By William Reiler, A. M., M. D. J. B. Lippencott & Co. Philadelphia, 1878.

Sub-Sulphate of Iron as an Antiseptic in the Surgery of the Pelvis. By H. P. C. Wilson, M. D. Baltimore, Md.

Medical Missions at Home and Abroad. By. J. G. Kerr, M. D.

Proceedings of the Medical Society of the State of Pennsylvania, in relation to the Hospital for Insane at Dixmont.

Notes from a Lecture. By Dr. Manuel Dagnino, of Caracas, on the treatment of Yellow Fever. Translated by Antonio de Tajada.

Removal of Naso-Pharyngeal Polypus by temporary depression of both upper jaws. By L. McLane Tiffany, M. D.

The Brain and Nervous System in relation to teaching and learning. By J. C. Reeve, M. D.

Variola; Its Causes, Nature and Prophylaxis, and the dangers of Vaccination. By C. Spinzig, M. D.

Treatment of Fracture of the Shaft of the Femur. By Franklin Staples, of Winona, Minn.

On the use of the Solid Rubber Bandage in Eczema and Ulcers of the Leg. By L. Duncan Bulkley, A. M., M. D.

On Diet and Hygiene in Diseases of the Skin. By L. Duncan Bulkley, A. M., M. D.

Annual Address delivered before the American Academy of Medicine. By Frank H. Hamilton, A. M., M. D., L. L. D.

Urethral Stricture. By Thomas Brown, M. D.

Address before the American Medical Association at Buffalo, N. Y. By T. G. Richardson, M. D.

Ecole de Medicine et de Chirurgie de Montreal.

Pocket Therapeutics and Dose-Book. By Joseph Stewart, Jr., B. A., M. D. Detroit, Mich.

THE BUFFALO

### MEDICAL AND SURGICAL

### MEDICAL AND SURGICAL JOURNAL.

PUBLISHED MONTHLY,—Containing Original Articles, Reports of Medical Societies and Hospitals, Editorial, Reviews, Correspondence, Army News, etc., etc; including the usual variety of Medical Periodical Publications. Specimen copies sent on application to BUFFALO MEDICAL AND SURGICAL JOURNAL, J. F. MINER, M. D., 978 Main Street, BUFFALO, N. Y.

TERMS OF SUBSCRIPTION. $3 00 PER YEAR, IN ADVANCE,—All communications for publication should be sent before the fifteenth (15) of each month, or they will of necessity lie over until the next number. No change can be made in advertisements after the twenty-fifth.

Correspondence and original articles are solicited. Publication is no evidence that the views of the author are endorsed by the Journal.

